# Premature Burial

**Published:** 1905-03-04

**Authors:** 


### EDITOR'S LETTER-BOX.

[Our Correspondents are reminded that prolixity is a great bar to publication, and that brevity of style and conciseness of statement greatly facilitate early insertion.]

### PREMATURE BURIAL.

In the course of a letter which we have not space to publish in full, Mr. James R. Williamson, 100 Chedington Road, Upper Edmonton, N., writes:—"If any reader of The Hospital would care to have a copy of the 'Signs and Proofs of Death,' by the late eminent physician, Sir B. W. Richardson, M.D., F.R.S., I should be pleased to send it on receiving a stamped envelope."

